
JOURNAL INFORMATION
==============================
NLM Title Abbreviation: Atlanta Med Surg J (1884)
NLM Journal ID: 101251623

Atlanta Medical and Surgical Journal (1884)

Publisher: Biomedical Journal Digitization Project

ARTICLE INFORMATION
==============================
PMCID: PMC9199291
PMID: 35829590
Article version: 1
Subjects: Correspondence

Postponement of the Third Pan-American Medical Congress

Print publication date: 1898 Dec
Volume: 15
Volume Series: New Ser
Issue: 10
First page: 679
Last page: 680
License: This work is free of known copyright restrictions. For more information, please see PMC Back Issue Digitization.
License URL: https://creativecommons.org/publicdomain/mark/1.0/

==============================

### CORRESPONDENCE.

### POSTPONEMENT OF THE THIRD PAN-AMERICAN MEDICAL CONGRESS.

INTERNATIONAL EXECUTIVE COMMISSION
OF THE PAN-AMERICAN MEDICAL CONGRESS.
OFFICE OF THE SECRETARY,
CINCINNATI, November 5, 1898.

MY DEAR SIR:—I have the honor to announce that in April, 1898, I received from Dr. José Manuel de los Rios, Chairman of the Committee on Organization of the 111 Pan-American Medical Congress, a request that, in consequence of the then existing rebellion in Venezuela, no definite arrangements be made at that time relative to the meeting of the Congress previously appointed to be held in Caracas in December, 1899.

The following communication relative to the same subject is just at hand:

CARACAS, September 25, 1898.

*Dr. Charles A. L. Reed, Secretary of the International Executive Commission, Cincinnati, Ohio:*

DEAR SIR:—After having sent my communication dated April last, I find it to be my duty to notify you that, although the considerations pointed out in it have already ended, our country has been scourged by smallpox which has taken up all our physicians' activities and time, depriving them of going into scientific works. And, as that state of mind of our people and government after such calamities as war and epidemic would greatly enterfere with the good success of our next meeting, I beg leave to tell you, in order you will convey it to the International Executive Committee, that our Government and this Commission would be grateful to have the meeting which was to take place in Caracas in December, 1899, adjourned for one year later. I am, dear Doctor,

Yours respectfully,

THE PRESIDENT.

[Signed.] DR. JOSÉ MANUEL DE LOS RIOS.

In accordance with the request of the Government of Venezuela, and of the Committee on Organization, the 111 Pan-American Medical Congress is hereby postponed to meet in Caracas in December, 1900.

For the International Executive Commission.

CHARLES A. L. REED,
Secretary.

---

***NOTICE.—This JOURNAL and THE INTERNATIONAL JOURNAL OF SURGERY one year for $2.00 cash.***

---

### FOR IVY POISONING.

Keep affected parts wet with freshly made lime-water. Give the following mixture internally:

℞ Ext. tritici fl........................................ʒ iv.
Spts. etheris nitrosi } aa.................................℥ i.
Syr. limonis }
M. Sig. One teaspoonful four times a day.—*Ex.*

---

### REMOVAL OF FRECKLES.

1. Apply the following lotion to the face morning and evening:

℞ Zinci sulphocarbolat ..................................ʒ i.
Glycerini..............................................℥ ii.
M. Sig. External use.

2. Cover the freckles every other day with the following ointment:

℞ Hydrarg. ammoniat } aa.................................ʒ i.
Bismuth subnitrat }
Glycerit amyl..........................................℥ ss.
M. Ft. ungt. Sig. External use.—*Tonvenaint—Ex.*

---

**NOTICE.—This JOURNAL and THE INTERNATIONAL JOURNAL OF SURGERY one year for $2.00 cash.**
